# Potential of Endogenous Oxytocin in Endocrine Treatment and Prevention of COVID-19

**DOI:** 10.3389/fendo.2022.799521

**Published:** 2022-05-03

**Authors:** Stephani C. Wang, Fengmin Zhang, Hui Zhu, Haipeng Yang, Yang Liu, Ping Wang, Vladimir Parpura, Yu-Feng Wang

**Affiliations:** ^1^ Division of Cardiology, Department of Medicine, University of California-Irvine, Irvine, CA, United States; ^2^ Department of Microbiology, School of Basic Medical Sciences, Harbin Medical University, Harbin, China; ^3^ Department of Physiology, School of Basic Medical Sciences, Harbin Medical University, Harbin, China; ^4^ Neonatal Division of the Department of Pediatrics, Harbin Medical University The Fourth Affiliated Hospital, Harbin, China; ^5^ Department of Genetics, School of Basic Medical Sciences, Harbin Medical University, Harbin, China; ^6^ Department of Neurobiology, The University of Alabama at Birmingham, Birmingham, AL, United States

**Keywords:** hypothalamus, neuroendocrinology, neurohumoral reflex, physiotherapy, prevention, SARS-CoV-2, treatment, virus

## Abstract

Coronavirus disease 2019 or COVID-19 caused by severe acute respiratory syndrome coronavirus 2 (SARS-CoV-2) has become a significant threat to the health of human beings. While wearing mask, maintaining social distance and performing self-quarantine can reduce virus spreading passively, vaccination actively enhances immune defense against COVID-19. However, mutations of SARS-CoV-2 and presence of asymptomatic carriers frustrate the effort of completely conquering COVID-19. A strategy that can reduce the susceptibility and thus prevent COVID-19 while blocking viral invasion and pathogenesis independent of viral antigen stability is highly desirable. In the pathogenesis of COVID-19, endocrine disorders have been implicated. Correspondingly, many hormones have been identified to possess therapeutic potential of treating COVID-19, such as estrogen, melatonin, corticosteroids, thyroid hormone and oxytocin. Among them, oxytocin has the potential of both treatment and prevention of COVID-19. This is based on oxytocin promotion of immune-metabolic homeostasis, suppression of inflammation and pre-existing comorbidities, acceleration of damage repair, and reduction of individuals’ susceptibility to pathogen infection. Oxytocin may specifically inactivate SARS-COV-2 spike protein and block viral entry into cells *via* angiotensin-converting enzyme 2 by suppressing serine protease and increasing interferon levels and number of T-lymphocytes. In addition, oxytocin can promote parasympathetic outflow and the secretion of body fluids that could dilute and even inactivate SARS-CoV-2 on the surface of cornea, oral cavity and gastrointestinal tract. What we need to do now is clinical trials. Such trials should fully balance the advantages and disadvantages of oxytocin application, consider the time- and dose-dependency of oxytocin effects, optimize the dosage form and administration approach, combine oxytocin with inhibitors of SARS-CoV-2 replication, apply specific passive immunization, and timely utilize efficient vaccines. Meanwhile, blocking COVID-19 transmission chain and developing other efficient anti-SARS-CoV-2 drugs are also important. In addition, relative to the complex issues with drug applications over a long term, oxytocin can be mobilized through many physiological stimuli, and thus used as a general prevention measure. In this review, we explore the potential of oxytocin for treatment and prevention of COVID-19 and perhaps other similar pathogens.

## 1 Introduction

Coronavirus disease 2019 or COVID-19 is caused by severe acute respiratory syndrome (SARS) coronavirus 2 (CoV) or SARS-CoV-2. It has high morbidity and mortality and constitutes severe threats to human healthcare system. According to the report of Worldometers (https://www.worldometers.info/coronavirus/) on March 7, 2022, the confirmed cases of COVID-19 reached ~447.4 million including ~60.5 million active cases and ~6.0 million deaths. SARS-CoV-2 appears highly contagious; severe COVID-19 has poor prognosis; its treatments largely resolve symptoms only. While vaccination is available, there has been a substantial anti-vaccination pushback. Thus, exploration of novel prevention and treatment strategies that are independent of vaccination becomes essential for conquering COVID-19. In this review, based on knowledge about the correlation between COVID-19 pathogenesis and endocrine disorders, we explore the therapeutic potential of oxytocin against COVID-19 and the prevention value of mobilizing endogenous oxytocin secretion.

### 1.1 Challenge for Prevention of COVID-19

SARS-CoV-2 virus belongs to the family of coronaviruses that can cause diseases like severe acute respiratory syndrome or SARS, and Middle East respiratory syndrome or MERS. It has many special features in the epidemiology and pathogenesis.

### 1.2 Epidemiology

Human-to-human transmission of SARS-CoV-2 has been confirmed. Small droplets and aerosols containing the virus can spread from an infected person’s nose and mouth as they breathe, cough, sneeze, sing, or speak. Recently, while bats are considered as the reservoir host, potential intermediate hosts are also identified such as pangolins (1) and tree shrews (2). In addition to the wild animals, angiotensin-converting enzyme 2 (ACE2), viral receptor proteins from dog, cat, and cattle are found to be the most permissive route to SARS-CoV-2 entry (3). Since these domestic animals have intimate contact with humans, the possibility for animal-to-human transmission cannot be excluded. Correspondingly, they should be monitored as potential intermediate hosts.

Compared to the youngsters, more severe diseases of COVID-19 are associated with elderly, immune-suppressed, and those who have existing co-morbidities such as coronary artery diseases, hypertension, and diabetes. However, young people are also a target of SARS-CoV-2, particularly among those who are unvaccinated. According to an investigation during May 1-July 25, 2021, among 43,127 reported SARS-CoV-2 infections in Los Angeles County residents aged ≥16 years, 30,801 (71.4%) were in unvaccinated persons, and 10,895 (25.3%) were in fully vaccinated persons. Much lower percentages of fully vaccinated persons infected with SARS-CoV-2 were hospitalized (3.2%) and were admitted to an intensive care unit (0.5%) compared with unvaccinated persons (7.6%, and 1.5%, respectively). However, the percentages of Delta variant infections estimated from 6,752 samples with lineage data increased among fully vaccinated persons (from 8.6% to 91.2%), and unvaccinated persons (from 8.2% to 87.1%) (4). Amidst circulation of the Delta variant, individuals aged 12 years and older in schools also become high-risk population (5, 6). Thus, COVID-19 targets all age groups, particularly the unvaccinated population with neonates as an exception (7).

Most people infected with the SARS-CoV-2 experience mild to moderate respiratory illness and recover spontaneously; however, elderly, and those who had underlying medical problems are more likely to develop serious conditions (8, 9). Thus, direct exposure to the virus, lack of vaccination and poor health conditions are the key factors determining individuals’ susceptibility to COVID-19.

### 1.3 Evaluation of Current Treatment and Prevention Measures

In the efforts of controlling COVID-19, while vaccination has been applied extensively, many drugs have also been explored (10, 11). Recently, molnupiravir has been considered as a breakthrough of COVID-19 treatment since it can reduce the risk of severe cases and mortality to 50% (12). Moreover, Pfizer reported that a 5-day paxlovid pill regimen to treat early infections with SARS-CoV-2 is 89% effective in patients at risk of serious illness (13, 14). Both drugs have gained emergency use authorization by the U.S. Food and Drug Administration [Coronavirus (COVID-19) Update: FDA Authorizes Additional Oral Antiviral for Treatment of COVID-19 in Certain Adults | FDA] (https://www.fda.gov/news-events/press-announcements/coronavirus-covid-19-update-fda-authorizes-first-oral-antiviral-treatment-covid-19). However, the effectiveness of molnupiravir and paxlovid are limited within a narrow window at the early stage of COVID-19. In addition, incomplete inhibition of viral replication and subsequent drug-resistance remain to be monitored. Importantly, some potentially effective drugs act as a double-edged sword, such as ACE inhibitor captopril that decreases tissue anti-inflammatory response while reducing viral entry (15). In addition, after controlling symptoms of severe COVID-19 patients during acute phase, long term sequelae remain a challenge for rehabilitation. Therefore, further clarification of the underlying mechanism and exploration of novel treatment and prevention strategies of COVID-19 are highly demanded.

Among current measures of prevention, passive prevention measures like wearing mask, social distancing and self-isolation could not provide sufficient protection (16) even for those who have received two doses of vaccination (4). It is true that vaccination can substantially reduce the morbidity and mortality at early stage of COVID-19 pandemic; however, when the dominant variants changed from Alpha to Delta and Omicron variants, the initial protective effect of vaccination droped dramatically (17–19). This is because Delta S protein can fuse membranes more efficiently at low levels of cellular receptor ACE2 and its pseudotyped viruses infect target cells substantially faster than all previous variants tested, thereby heightening its transmissibility (20). Similarly, passive immunization by giving antibody isolated from the plasma of recovered patients to another individual who is at risk of infection can provide instant, short-term fortification against infectious agents and slow down the spread of COVID-19 (21, 22). Recent construction of neutralizing nanobodies that target the receptor binding domain of the SARS-CoV-2 spike (S) protein seems more effective that natural antibodies against COVID-19 (23), albeit this remains to be tested in clinical setting.

The latest SARS-CoV-2 variant Omicron (B.1.1.529) has posed further challenges for the anti-COVID-19 efforts due to its contagiousness and vaccine-escape mutations, the latter involving not only nucleotide substitutions and short deletion but also insertions in the S protein that are predicted to lead to escape from neutralizing antibodies and from T-cell immunity. Omicron variant seems to be ten times more contagious than the original virus, about twice as infectious as the Delta variant, twice more likely to escape current vaccines than the Delta variant, and previous effective antibodies may be seriously compromised (24, 25). **Table 1** shows SARS-CoV-2 variants classification and virulence.

**Table 1:** SARS-CoV-2 variants classification and virulence.

WHO Label	Pango Lineage	*Date of Designation	Spike Protein Substitutions	First Identified	**Attributes	References
Alpha	B.1.1.7 and Q lineages	VOC: December 18, 2020	N501Y, HV 69/70 deletion, P681H	UK in September 2020 and major variants in the first half year of 2020	Associated with increased transmissibility and risk of death compared with other variants.	(22, 26)
Beta	B.1.351 and descendent lineages	VOC: December 18, 2020	Multiple mutations at K417N, E484K, N501Y	South Africa May, 2020	Low transmissibility, high immune evasiveness; E484K, may affect neutralization by some polyclonal and monoclonal antibodies	(27, 28)
Gamma	P.1; a branch off the B.1.1.28 lineage	VOC: January 11, 2021	Multiple mutations at K417T, E484K, N501Y	Brazil in November, 2020. The biggest threat to the outbreak in South America; there is also a risk of secondary infection.	Affect its transmissibility and antigenic profile.	(29)
Delta	B.1.617.2 and AY lineages	VOC: May 11, 2021	T19R, (V70F*), T95I, G142D, E156-, F157-, R158G, (A222V*), (W258L*), (K417N*), L452R, T478K, D614G, P681R, D950N	India in October, 2020 and then most countries in the world.	Increased transmissibility; nearly all lineages designated as Delta are susceptible to Emergency Use Authorization monoclonal antibody treatments except AY.1 and AY.2 lineages.	(30)
Omicron	B.1.1.529 and BA lineages	VOC: November 26, 2021	A67V, del69-70, T95I, del142-144, Y145D, del211, L212I, ins214EPE, G339D, S371L, S373P, S375F, K417N, N440K, G446S, S477N, T478K, E484A, Q493R, G496S, Q498R, N501Y, Y505H, T547K, D614G, H655Y, N679K, P681H, N764K, D796Y, N856K, Q954H, N969K, L981F	South Africa in November, 2021 and then spreading to multiple countries.	Transmission and replacement of the Delta variant in South Africa. Have both the features of Beta in high immune evasiveness and of Delta in high transmissibility. Relative to Delta, there are >20% reduction in morbidity and >35% reduction in admission to hospital.	(31, 32)

VOC, Variant of Concern; * refers to https://www.who.int/en/activities/tracking-SARS-CoV-2-variants/; ** refers to https://www.who.int/health-topics/coronavirus#tab=tab_.

Although new vaccine and treatment efficiency remains to be tested, the continuous passive defense against COVID-19 reaches a turning point for seeking more stable protective measures. Correspondingly, prevention of COVID-19 should depend not only on current passive measures of prevention and vaccination, but also on boosting individuals’ immunity against COVID-19 while developing more effective treatment strategies.

### 1.4 Pathogenesis

Understanding of COVID-19 pathogenesis is the prerequisite to treat and prevent COVID-19. Viral entry into host cells is mainly mediated by the binding of its S protein to ACE2 receptor (33). The activation of S protein requires the cleavage of a transmembrane serine protease 2 (TMPRSS2) (34) and furin (35) on ACE2-positive cells. ACE2-positive cells include lung alveolar epithelial cells, enterocytes of the small intestine, renal tubules, heart, arterial and venous endothelial cells, arterial smooth muscle cells, beta cells, cerebral neurons (36–38), as well as monocytes, macrophages and lymphocytes (39). In this process, binding of viral S protein to heparan sulfate proteoglycans (HSPGs) leads to the enrichment of local tissue concentration of SARS-CoV-2 for the subsequent specific binding with ACE2. HSPGs assist ACE2 in endocytosis-mediated SARS-CoV-2 cellular internalization by transiting the virus from the extracellular environment to the inside of cells (40, 41). The endocytosis is clathrin-dependent with virus sequestered into an endosome (42). In the endosome, the lysosomal endopeptidase cathepsin B/L can facilitate the fusion of virus with endosomal membranes. SARS-CoV-2 then exploits the endogenous transcriptional machinery of infected cells to replicate and spread through the entire organ (43). Many cellular host factors and the innate immune cells act to inhibit virus entry and intracellular replication through pattern-recognition receptors and intracellular RNA sensor molecules (44, 45). However, the non-structural proteins of the virus can hijack them by recruiting host partners to form hetero-oligomeric complexes and then suppress interferon synthesis and its antiviral effects (46). Thus, multiple cellular components are involved in the invasion of SARS-CoV-2 and they can be the targets for blocking SARS-CoV-2 infection.

After infecting target cells, SARS-CoV-2 completes its own replication inside of the cell and then releases itself outside of the cell, thereby destroying the old cells and infecting new ones. In this process, the amount of cell partitioned ACE2 is also reduced, which results in an elevation of angiotensin II concentration in the blood and inflammation (47). In response to viral attacks, the body first initiates an innate immune antiviral response by recruiting neutrophils and macrophages while releasing cytokines. Then, by activating and recruiting lymphocytes, the body initiates an adaptive immune response that plays a specific antiviral role. In severe cases, particularly in the elderly, the virus can interact with the immune system to induce an excessive innate immune response, or even a cytokine storm, which can disrupt the epithelial cells in the lung and the gas-blood barrier, leading to the acute respiratory distress syndrome (ARDS). At the same time, the virus also attacks lymph tissue and then causes lymphopenia that prevents the virus from clearance (48–50). Correspondingly, relative lymphopenia, cytokine storm, and multiple organ failure become significant indicators of COVID-19 severity and therapeutic targets.

## 2 COVID-19 and Endocrine Activity

In the pathogenesis of COVID-19, endocrine disorders are a significant feature (51). Studies have revealed that hypothalamus, pituitary, pancreas, thyroid, adrenal glands, testes, and ovaries all express ACE2 (52). Thus, hormones produced in these endocrine organs could all be influenced. In COVID-19 patients, hypothalamic damage with SARS-CoV-2 has been identified, which suppresses hypothalamic neuroendocrine activity (53), similar to some other viral diseases (**Table 2**). The neural injury caused by SARS-CoV-2 is associated with increased activity of the TMPRSS2 and cathepsin L and secondary increase of angiotensin II following downregulation of ACE2. Resultantly, upregulation of proinflammatory mediators and reactive oxygen species occurs, which causes neuroinflammatory response and blood brain barrier disruption. Furthermore, dysregulation of hormone and neurotransmitter signaling may constitute a fundamental mechanism involved in the neuropathogenic sequelae of SARS-CoV-2 infection (71). Thus, endocrine disorders are present in COVID-19 patients and can play important roles in the development of COVID-19 pathogenesis and prognosis.

**Table 2:** Effects of viral infection on the oxytocin system, and general antiviral properties and experimental evidence supporting oxytocin anti-COVID-19 functions.

Items	Entities	Conditions or OT/OTR signaling	Target (effects)	Outcomes	References
Effects of viral infection on the OT system	HIV patients	OT immunoreactivity in hypothalamic neurons	OT cells in the hypothalamus (reduction)	COVID-19-like immune disorders	(54)
	SARS patients	Detection of SARS viral particles and genomic sequence SARS-CoV	Hypothalamus and the cortex (high levels)	Neural and endocrine disorders	(55)
	COVID-19 patients	Whole-brain voxel-based analysis of (18)F-FDG PET metabolism	Metabolic changes in the hypothalamus (low levels)	Reduction of associated endocrine activities.	(56)
	Women living with HIV	Low levels of OT, stress status, CD4+ cell counts	Association between stress and CD4+ cell counts (inverse)	Stress and low immunity	(57)
	Mice	Effects of HIV-1 Tat on OT levels in several brain regions and PVN OT neurons	The prefrontal cortico-hippocampalamygdala circuit (reduction)	Aberrant social behavior	(58)
	Mice	Maternal exposure to influenza A virus (H3N2) on offspring health	OT and serotonin levels in the brain (Reduction)	Disorders in social behaviors	(59)
OT antiviral functions	Mouse spleen cell cultures	OT effect on INF-γ expression	INF-γ reduces cathepsin L, but induces antiviral protein Mx	Restrict enveloped RNA viruses entry	(60, 61)
	High throughput screening	HP-3, an OT antagonist, directly interacts with HCV-IRES	HCV-IRES recruits eukaryotic translation initiation factor 3	Increase replication of HCV and translation	(62)
	Cows	Metritis, postpartum uterus involution and multiparity increase OTR expression	Increase antiviral factor Mx2 and MYH10 gene	Increase macrophages recruitment	(63)
	Human amnion cells	OT causes association of protein kinase C activity to the membrane fraction	Activate downstream pathway of interferon	Inhibit stomatitis virus multiplication	(64)
Anti- SARSCoV-2 effects	Humans	OT inhibits dipeptidyl peptidase-4	Suppress SARS-CoV2 entry of human respiratory tract	Inhibit viral entry and severe COVID-19	(65, 66)
	Transcriptomic signature, cell line	Carbetocin more effectively induces immune cell responses than either lopinavir or hydroxychloroquine, COVID-19 drugs	Reduce inflammation and T cell inhibition, and enhance T cell activation	Suppress major immune disorders in COVID-19 patients	(67)
	Drug screening and in Silico modeling	OT dose-dependently inhibits the binding of recombinant, trimeric SARS-CoV-2 spike protein to recombinant human ACE2.	SARS-CoV-2 entry of ACE2- expressing cells	COVID-19 treatments or prophylactics	(68)
Alleviating COVID-19 symptoms	Pregnant woman	Exposing to SARS-CoV-2 at the 3^rd^ stage, giving birth after 37 weeks to a neonate, and receiving OT treatment after delivery	The neonate has positive immunoglobulin G and negative nucleic acid tests	Both mother and infant recovered well	(69)
	Rats with sepsis-induced acute lung injury	OT reduced increased lactic acid, C-reactive protein, IL-6, tumor necrosis factor alpha, interleukin IL 1β levels	Lung and the immune system	Protective effect of OT in ARDS	(70)

ACE2, angiotensin-converting enzyme 2; ARDS, Acute respiratory distress syndrome; HCV, hepatitis C virus; IRES, internal ribosome entry site; HIV, human immunodeficiency virus; IL, interleukin; INF-γ, interferon-γ ; OT, oxytocin; OTR, OT receptor; PVN, paraventricular nucleus; SARS-CoV2, severe acute respiratory syndrome coronavirus 2.

### 2.1 Activity of the Hypothalamic-Pituitary-Endocrine Gland Axes

#### 2.1.1 Hypothalamic Pituitary Adrenal (HPA) Axis

Hypofunctions of the HPA axis have been identified in COVID-19 patients and corticosteroids can effectively alleviate severe ARDS (72). When COVID-19 is more severe, the patients have lower cortisol and adrenocorticotropic hormone levels (73), suggesting a direct link between the COVID-19 infection and impaired glucocorticoid response. However, there is no evidence showing reduced corticotropin-releasing hormone secretion, the top regulator of the HPA axis, and the causal association between the reduced HPA axis activity and COVID-19 severity. In alleviating syndrome of COVID-19, the usage of glucocorticoid hormones can inhibit inflammation and cytokine storm and thus, improve the condition of severe COVID-19 patients by (re)activating ACE2 and reducing interleukin (IL)-6 levels (74). Notably, although the HPA axis activity is deviated during COVID-19, dexamethasone treatment is not applied to simply compensate the insufficiency of corticosteroid production. Dexamethasone is administered to patients under oxygen treatment and having inflammatory symptoms to modulate immune response rather than supplementing basal corticosteroid demand. Glucocorticoid also reduces body’s resistance to viral infection and increases stress reaction (75). Thus, despite that the application of corticosteroids promotes patient’s short-term physical recovery, such as improving hypotension and significant hyponatremia (76), survivors of critical illness have significantly elevated risk of developing lasting cognitive impairment and psychiatric disorders (77).

#### 2.1.2 Hypothalamic-Pituitary-Thyroid Axis

Similar to the HPA axis, there is also evidence that suggests reduced activity of the hypothalamic pituitary thyroid axis. The levels of total triiodothyronine (T3) and thyroid stimulating hormone (TSH) are lower in COVID-19 patients (78, 79). It was also reported that within the group of COVID-19 cases, 61.9% (52/84) patients presented with thyroid function abnormalities and the proportion of thyroid dysfunction was higher in severe cases than mild/moderate cases. Patients with thyroid dysfunction tended to have longer viral nucleic acid cleaning time in association with decreased number of lymphocytes and increased level of C-reactive protein (80). A positive correlation between low mean T3 level and clinical severity of COVID-19 was also reported (81). Consistently, in six articles qualified for the final analysis which included 1160 patients, most of the patients had lower mean T3 level and normal or low TSH level. Clinically, thyroid hormones may relieve hypothalamic hypometabolism in COVID-19 patients (53). However, the complex interplay between thyroid hormone action and the immune system is still not completely understood (82).

#### 2.1.3 Hypothalamic-Pituitary Gonad Axis

Relative to other hormones, the involvement of sex hormones in COVID-19 is more complex. Men have a higher risk of death from COVID-19 than women and androgens may facilitate entrance of the SARS-CoV-2 virus into respiratory epithelial cells (83, 84). Consistently, that androgens promote transcription of TMPRSS2 (85, 86) seems to suggest that higher testosterone levels promote SARS-CoV-2 entry into the cells. Correspondingly, androgen deprivation therapy reduces infection rates and improves outcomes for COVID-19 (87).

However, there is an increased COVID-19 case fatality in the hypogonadotropic hypogonadism cohort, particularly in aged men; this perhaps reflects an underlying pro-inflammatory state (88). Namely, testosterone can suppress both the cellular and humoral immune systems such as lowering IL-6 and tumor necrosis factor-α levels *via* inhibition of the NF-κB proinflammatory pathway. Low levels of testosterone in aging men are linked with high inflammatory markers such as IL-6 and increased risk of lung damage after pneumonia (89). In addition, androgen deprivation therapy has been associated with adverse cardiovascular events (90), endothelial dysfunction, thrombosis and defective immune response (91). Consistently, the testicle expresses high levels of ACE2, making it a target of SARS-CoV-2, which could account for the low testosterone and high luteinizing hormone levels in COVID-19 males (86). Thus, application of testosterone to COVID-19 patients should be performed with caution.

In contrast to androgens, estrogens produce pro-inflammatory effects. Estrogens increase the immunological defense against pathogens while estradiol can down-regulate ACE2 in the airway epithelium (92), which may reduce viral invasion and make estrogens a good candidate for increasing the immunity against COVID-19 (93). However, estrogens may promote neutrophilic inflammation in subjects with asthma and chronic obstructive pulmonary disease. Age-associated decreases in estrogen may also mediate proinflammatory reactions that could increase patient's risk of COVID-19 adverse outcomes (89). Thus, estrogen may be effective for postmenopausal women but not suitable for women with menstruation who have periodic fluctuations in estrogen versus progesterone levels (94). In addition, hormone replacement therapy in aged women has been found to possess cancer-evoking effect (95). Thus, estrogen cannot be used as a regular agent against COVID-19.

### 2.2 Neurohypophysial Hormones

**Figure 1:**
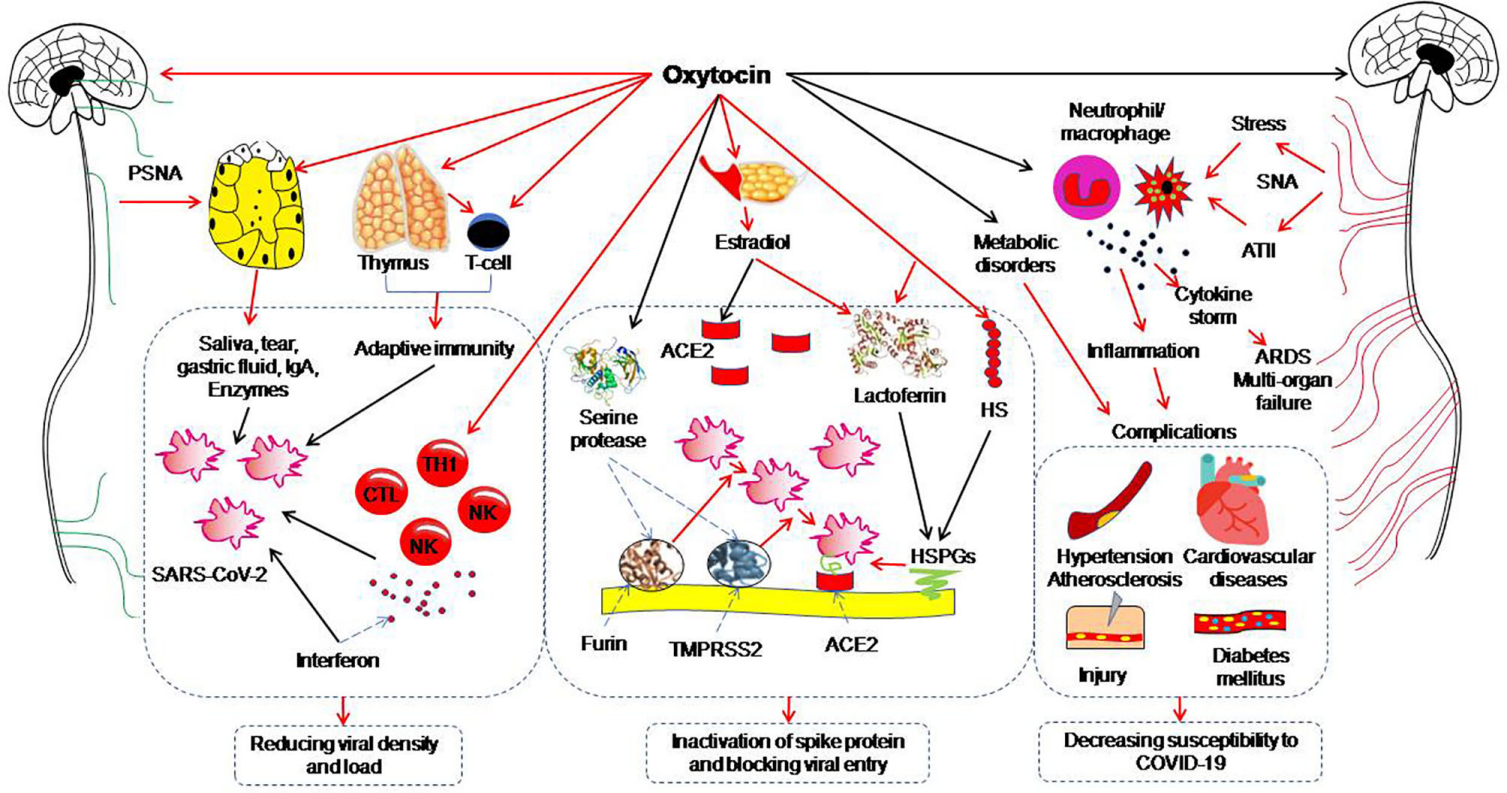
Potential preventive functions of oxytocin against coronavirus disease (COVID-19). The red arrows indicate direct activation; the black arrows indicate direct inhibition. ACE2, angiotensin-converting enzyme 2; ARDS, acute respiratory distress syndrome; ATII, angiotensin II; CTL, cytotoxic T lymphocyte; HS, heparan sulphate; HSPG, heparan sulphate proteoglycan; IgA, immunoglobulin A; NK, nature killer cell; PSNA, parasympathetic nerve activation; SARS-CoV-2, severe acute respiratory syndrome coronavirus 2; SNA, sympathetic nerve activation; TH1, T helper cells 1; TMPRSS2, Transmembrane protease serine 2. [Figure is original drawing; also refer to references (96, 100–102)].

The neurohypophysial hormones include oxytocin and vasopressin. Oxytocin, a classical hypothalamic neuropeptide mainly produced in the supraoptic nucleus and paraventricular nucleus, carries functions in immunologic defense, homeostasis and surveillance, has the properties of anti-virus, anti-inflammation, antibiotics, and cell protection (96, 97) and thus, has the potential to antagonize COVID-19 (**Table 2**). This view is supported by the following studies. The transcriptomic signature of carbetocin, an oxytocin agonist, has a pattern of concordance with inflammation (i.e. IL-1β and IL-6) and immune marker knockdown signatures that are consistent with reduction of inflammation and promotion of immune response (i.e. T cell and macrophage cell markers like CD40 and ARG1). Importantly, carbetocin is more effective at inducing immune cell responses than either lopinavir or hydroxychloroquine, both of which have been explored for the treatment of COVID-19 (67). Consistently, in screening 2,701 compounds from a commercial library of drugs that potentially block the interaction between the S protein and ACE2, oxytocin is among four best candidates along with thiostrepton, nilotinib, and hydroxycamptothecin (68). This proposal is also in agreement with the view that oxytocin may be repurposed as an agent for treatment of the COVID-19 patients (98–100). Overall, these studies highlight beneficial potential of oxytocin for treating COVID-19 patients. **Figure 1** illustrates a variety of oxytocin anti-COVID-19 functions.

Along with oxytocin, vasopressin is also implicated in the treatment of COVID-19. Vasopressin plays a key role for the maintenance of osmotic, cardiovascular, and stress hormone homeostasis during many diseases. Activation of the vasopressin system is a common finding of respiratory infectious diseases; a pronounced activation of the vasopressin system in COVID-19 patients is associated with an adverse clinical course in COVID-19 patients (103). By contrast, vasopressin is commonly used in COVID-19 respiratory failure. This is because vasopressin infusion reduces total norepinephrine requirements for maintaining blood pressure while sparing the pulmonary vasculature (104). COVID-19 patients with respiratory failure experience hypoxic pulmonary vasoconstriction while vasopressin produces less right heart strain than catecholamine. Interestingly, some of these vasopressin functions could be mediated by oxytocin receptor (OTR). For example, in septic shock, vasopressin is commonly used as a vasopressor to restore blood pressure while having anti-inflammatory effects as well. Vasopressin decreases the responsiveness of human aortic endothelial cells to tumor necrosis factor-α by inducing a disintegrin and metalloprotease 10-dependent ectodomain shedding of tumor necrosis factor receptor 1. These effects of vasopressin are blocked by blocking OTR activation (105). This finding again highlights the importance of oxytocin in controlling COVID-19.

The SARS-CoV-2 S protein, soluble ACE2, and vasopressin can form molecular complexes that facilitate cellular infection through endocytosis mediated by vasopressin receptor-1b, and thus vasopressin treatment could promote cellular infection and systemic viral dissemination (106). Therefore, while vasopressin infusion in COVID-19 is beneficial for critical illness, its potential for increasing viral infection limits its regular clinical application.

### 2.3 Others

In addition to the hypothalamic-pituitary hormones, many other hormones are also implicated in COVID-19 pathogenesis. It is well established that the pathology of COVID-19 is strongly related to the renin-angiotensin system, particularly angiotensin II. Cell surface ACE2 is largely lost following SARS-CoV-2 invasion, which destroys infected cells while reducing ACE2 expression and activity. As a result, angiotensin II could not be fully converted to inactive heptapeptide of the angiotensin 1-7 by the remaining ACE2, which increases blood angiotensin II levels (47). Increased levels of angiotensin II can cause neutrophil accumulation, vascular hyper-permeability, and pulmonary edema (107). By contrast, ARDS patients receiving infusion of ACE2 manifest decreased angiotensin II and IL-6 levels, and increased surfactant protein (108). Thus, reducing ACE2 loss and suppression of angiotensin II activity are considered in controlling COVID-19 development (109). However, this measure potentially increases individuals’ susceptibility to re-infection of SARS-CoV-2.

Another important hormone is melatonin. Melatonin has anti-inflammatory and anti-oxidative effects and is protective against ARDS caused by viral and other pathogens. Thus, melatonin may be effective in critical care patients by reducing vessel permeability, anxiety, sedation use, and improving sleep quality (110). Notably, endogenous melatonin is produced in dark but, as social creatures, people cannot stay in dark while maintaining normal social activity. Thus, mobilization of melatonin is not suitable as a prevention measures against COVID-19.

## 3 Therapeutic and Preventive Potential of Oxytocin Against of COVID-19

As stated above, increasing density of virions in the body and their entry into the cells are the initial step of COVID-19 pathogenesis, while poor body resistance to viral invasion is the key mechanism. Thus, in addition to the measures of avoiding SARS-CoV-2 contamination, improving immunological condition, reducing the number of virions in the body and blocking viral entry into the cells should be considered. Oxytocin is a strong candidate to carry out these missions through multiple approaches (**Figure 1**). **Table 2** summarizes the effects of viral infection on the oxytocin system, its general antiviral properties and experimental evidence supporting oxytocin anti-COVID-19 functions.

### 3.1 Oxytocin and the Pathogenesis of COVID-19

In the pathogenesis of COVID-19, oxytocin neuronal activity is inhibited. In those who are susceptible to COVID-19, such as menopausal women (111, 112), aged man (113) and patients with chronic diseases such as diabetes (114), oxytocin secretion is reduced. In COVID-19 patients, hypothalamic neural activity is low (53, 115), likely because of olfactory bulb-mediated infection of the hypothalamus (116, 117). Consistently, COVID-19 patients have lower circulating levels of natriuretic peptides (118) that are under facilitatory regulation of oxytocin (119). The increased angiotensin II due to reduction of ACE2 (120) can also reduce circulating oxytocin levels (121). Consequently, the insufficiency of oxytocin in the brain and blood can make individuals fall in panic, fear and immune-metabolic disorders (122, 123), thereby reducing resistance to COVID-19. Thus, enhancing basal oxytocin neuronal activity and restoring its activity in COVID-19 patients appear critical for controlling the development of COVID-19 pathogenesis as well as prevention of COVID-19.

### 3.2 Decreasing Susceptibility to COVID-19

Oxytocin, based on its extensive immune-regulating properties (97), has recently been recommended as a candidate to treat pathogenesis of COVID-19 (98, 99, 124). This is because oxytocin can suppress cytokine storm, improve lymphocytopenia, prevent thrombosis, and avoid the occurrence of ARDS and multiple organ failures. Moreover, oxytocin has the potential to reduce COVID-19 susceptibility.

COVID-19 has high morbidity and mortality among elderly and individuals having immune-suppressed, and having existing co-morbidities such as coronary artery diseases, hypertension, diabetes and respiratory system disease (113, 125–127). These basal pathological conditions not only reduce individual’s general resistance to COVID-19 but also increase expressions of ACE2 and serine protease (33, 128), and thus increase body’s susceptibility to COVID-19. Correspondingly, improving immunological conditions and reducing these co-morbidities become necessary to reduce the morbidity and mortality of COVID-19. Oxytocin can suppress hyperlipidemia and hyperglycemia (123) that underlie obesity, atherosclerosis, diabetes and their associated diseases, such as hypertension, coronary artery disease and ischemic stroke (129). Oxytocin has immune-regulating functions (96, 102) and thus makes the body ready to fight against viral infection. Oxytocin has anxiolytic and anti-stress functions (130, 131), which, together with the anti-atherosclerosis effect, exerts anti-hypertension effect. In addition, oxytocin has also the potential to reduce cardiovascular complications and promote tissue regeneration (132). By prevention of these co-morbidities, oxytocin may reduce individuals’ susceptibility to COVID-19.

### 3.3 Reducing Viral Density/Number on the Surface of Target Cells

The onset of COVID-19 depends on the number of virions and their opportunity to bind with ACE2 while the presence of mucus and secretory fluids forms a natural barrier to separate virus from ACE2. The secretion of tears, salivary fluid, and gastric acid is mainly regulated by parasympathetic nerve activity. Increased parasympathetic nerve outflow promotes the secretion of these fluids and thus, can dilute the density of these virions. In addition, immunoglobulin and enzymes in some of these fluids also exert innate anti-viral functions. For example, in seventeen COVID-19 patients, all tear samples showed negative results of SARS-CoV-2 while nasopharyngeal swab samples continued to show positive result (133). It is likely that the lysozyme and immunoglobulin in tears have virus-killing effects (134). In addition, patients who use proton pump inhibitors have significantly higher morbidity of COVID-19 (135), indicating that gastric acid has sterile effect on SARS-CoV-2. Importantly, oxytocin can promote the activity of parasympathetic nerves, particularly the vagus (136), and thus indirectly facilitate the secretion of these body fluids and help remove and weaken the infection by SARS-CoV-2.

### 3.4 Inhibiting the Activation of S Protein and the Viral Entry Into the Cells

As stated above, the entry of SARS-CoV-2 into the cells relies on activation of the S protein by serine proteases, binding of the virus to the cell plasma membrane and endocytosis of viral particles. Oxytocin could interrupt these processes through several approaches, such as blocking the binding of SARS-CoV-2 to HSPG by stabilizing heparan sulphate and inhibiting the activity of serine protease like dipeptidyl peptidase-4 (65). Another potential approach of oxytocin against COVID-19 through HSPGs is its influencing the expression of lactoferrin that can bind to HSPGs and block viral attachment to the host cell (137). In addition to immunoglobulin A, lactoferrin is a natural anti-viral protein in milk (138, 139). The promotion of milk ejection by oxytocin is a natural venue to increase delivery of lactoferrin and immunoglobulin A to babies, and thus increases the defense of babies against COVID-19. This may account for why COVID-19 in neonates is uncommon and the majority of them have either asymptomatic infections or mild disease if infected (7), in which the action of oxytocin is at least a contributing factor.

Moreover, oxytocin has the potential to block the viral entry directly or through regulating the secretion of other hormones. As stated above, oxytocin is one of the four compounds that strongly inhibit binding of SARS-CoV-2 to ACE2 (68). Oxytocin may reduce viral entry through influencing estrogen secretion (94). There is a high rate of asymptomatic infection in pregnant women (140) because of a marked down-regulation of the expression of ACE2 in the airway epithelium (92) and TMPRSS2 (141) and activation of other protective functions (93). By increasing estradiol levels (142), oxytocin can decrease SARS-CoV-2 tropism following reduction in the expression of viral cell entry factors.

Oxytocin can also reduce viral entry through other approaches. Cigarette smoke causes a dose-dependent upregulation of ACE2 in a subset of secretory cells in the respiratory tract in rodent and human lungs (143). Intranasal administration of oxytocin reduces smoking craving (144) and thus may reduce the susceptibility to COVID-19. In addition, oxytocin can accelerate wound healing such as the lesions of skin (145), stomach (146), and intestine (147), and thus can strengthen the barrier against viral invasion. As a whole, oxytocin potentially suppresses the activation of S protein and viral entry into ACE2-positive cells.

### 3.5 Prevention of Psychophysical Disorders

During COVID-19 pandemic, mental health is a significant concern. Lockdown, quarantine measures, and social distancing are associated with increased depression, anxiety and distress during the pandemic (148). These psychological problems adversely affect immune functions and increase the severity of COVID-19-associated disorders and death rates (149). The perception of stress, isolation, and guilt among younger people was associated with poor mental health outcomes during COVID-19 (150, 151) and greater COVID-related social risk-taking behaviors, such as making and visiting new friends in person (152). Thus, social stress can worsen the pathogenesis in COVID-19 patients while increasing individuals’ susceptibility to SARS-CoV-2 infection.

In relieving COVID-19-related social stress, oxytocin is clearly a strong candidate. Stress-related to social isolation leads to downregulation of OTR in the ventral striatum in association with subjective distress and touch starvation (153). Oxytocin has anxiolytic and anti-stress functions, and thus can reduce the adverse neuroendocrine, autonomic, and behavioral responses (111). For instance, sexual intimacy involves increased oxytocin-associated activity in the limbic structures, nucleus accumbens, anterior cingulate, and prefrontal cortex, which help reduce stress and anxiety as well as their associated psychoneuroimmunity (154). Together with views of other researchers (155, 156), we believe that during the COVID-19 pandemic, activation of the endogenous oxytocin system or intranasal application of oxytocin should help resolve social isolation-induced inactivation of the oxytocin system and psychophysical disorders.

### 3.6 Potential Approaches of Applying Oxytocin to Treat COVID-19

Currently, there is one clinical trial that has been listed on https://clinicaltrials.gov first on May 13, 2020. It is a “Phase II RCT to Assess Efficacy of Intravenous Administration of Oxytocin in Patients Affected by COVID-19” (NCT04386447), carried out by CNRS, France, Azienda Ospedaliero Universitaria di Parma and Ospedale San Francesco Nuoro, Italy. Aiming to assess the effects of oxytocin in addition to standard therapy in reducing the number of patients who enter a critical stage, it proposed that “Intervention in addition to standard treatment, patients in the experimental arm will receive intravenous oxytocin with dilution of 25 IU or 40 IU oxytocin in 500cc physiological solution NaCl 9%. Oxytocin will be administered with continuous pump infusion with 62.5 ml/h. The clinician will have the option to decrease infusion speed based on significant variations in arterial pressure, otherwise, the total 25 IU or 40 IU amount will be infused in 8 hours. Treatment duration will be 10 days.” Currently, it is in withdrawn status since this study was not approved by the Italian Medicines Agency. Thus, presently there is no ongoing registered clinical trial of using oxytocin to treat SARS-CoV2 infections independently or along with other therapies.

However, clinical trials of oxytocin for COVID-19 treatment remain needed. Different approaches of oxytocin administrations and their advantage versus limitations have been considered (98, 100, 157, 158). These approaches mainly include intravenous and intranasal application of oxytocin, and optimal therapeutic usages have been tested experimentally. In an established murine model of chronic psychosocial stress, exposure to chronic subordinate colony housing results in long-lasting increase in anxiety, adrenal hypertrophy and thymus atrophy. Acute oxytocin administration in rodents can dose-dependently improve the behavior and physiology of male mice. However, chronic intracerebroventricular oxytocin infusion at high dose (10 ng/h) induces an anxiogenic phenotype with a concomitant reduction of OTR binding within the septum, the basolateral and medial amygdala, as well as the median raphe nucleus (159). Thus, chronically continuous application of oxytocin is not a preferred approach for treating COVID-19 patients. Alternatively, a single intranasal application of 26 IU of oxytocin causes a substantial rise of oxytocin plasma levels 30 min after intranasal administration in human participants; however, group mean oxytocin plasma level returns to baseline at 90 min post administration (160). Thus, it seems a preferred approach of intranasal oxytocin administration, particularly using dry inhaler but not in liquid spray (158), in an interval of 90 min for COVID-19 patients. This can be used in combination of molnupiravir and paxlovid programs. What we need urgently now is to initiate clinical trials and fully investigate the optimal doses and durations of treatment measures as Carter proposed (157).

## 4 Methods of Mobilizing Endogenous Oxytocin

Clinical drug use involves a large number of clinical trials, time, dose and dosage form, and users also need a large number of clinical observations. Certainly, new trials can be performed after carefully designing the research protocols; however, the development of COVID-19 cannot wait for a trial result. Thus, seeking the approaches of utilizing oxytocin to prevent individuals from COVID-19 and its serious pathological sequelae is necessary and urgent. Oxytocin can be mobilized endogenously through simple physical stimuli via the following approaches.

### 4.1 Vagal Stimulation

Oxytocin secretion can be evoked not only by the classic neuroendocrine reflex including the milk letdown reflex (161) and Ferguson reflex (162), but also their associated physical stimulation, particularly vagal stimulation.

Vagal activation is associated with distension of the gastrointestinal tracts, reduction of heart rate and blood pressure. Physiologically, distension of the gastrointestinal muscle layers by food sends afferent signals to the brainstem and reflex relaxation of the gastrointestinal tracts (163). Meanwhile, the afferents of vagal signals also reach the hypothalamus to increase oxytocin release into the blood (164) by the mediation of the vagus (165). Alternatively, probiotics in the gastrointestinal tracts can significantly increase blood oxytocin levels (166) by the mediation of the vagus (167). Thus, activation of vagal inputs can promote oxytocin secretion through feeding appropriately.

### 4.2 Activation of Oxytocin Neurons by Stimulating Special Sensory Organs

Oxytocin-mediated milk-letdown reflex can be conditioned around breastfeeding (168). In non-lactating females and men, activation of milk-letdown reflex-associated neural signals can elicit oxytocin release (169). The letdown reflex is critically dependent on the hypothalamic oxytocin neurons (161) that have extensive neural connection with specific sensory organs in the head. Thus, hypothalamic oxytocin neurons can receive signals from the olfactory bulbs, auditory cortex, retina, the medial frontal cortex and the mammillary body (170). For instance, aromatherapy with Rosa damascena can reduce the severity of pain and anxiety in the first stage of labor by increasing oxytocin release (171, 172). It has also been reported that baby’ cry can trigger the letdown reflex (173). In addition, listening to music (174), and shining light (175, 176), and even brief meditation (177) can increase oxytocin release independent of breastfeeding. Thus, activation of specific sensory organs and even mediation can increase oxytocin release.

### 4.3 Sexual Stimulation

It is well established that oxytocin release is increased significantly during sexual arousal, intercourse and orgasms for both men and women (178, 179). Thus, in addition to the Ferguson reflex that occurs when cervix is expanded during labor, reflex-release of oxytocin can also be evoked by genital organ stimulation (180, 181). The mechanical stimulation can activate oxytocin neurons in the hypothalamus, and thus increase oxytocin release from the posterior pituitary (182). Interestingly, vaginocervical stimulation can activate oxytocin neurons and their secretory activity by activating the vagus nerves (183, 184). Thus, sexual stimulation in adults is a way to increase endogenous oxytocin secretion.

### 4.4 Low Force Soothing Stimulation

In addition to vagal stimulation, modulating activity of special sensory organ, conditional reflex and sexual stimulation, oxytocin secretion can be facilitated by physical stimuli featuring low force, low frequency and slow conduction. In the assistant techniques for parturition, the combination of breathing exercises, foot reflexology and back massage can facilitate labor of women by reducing stress, pain, anxiety and promoting cervix ripening *via* increasing oxytocin release (185, 186). Stimulation with acupuncture at SanyinJiao can increase the release of oxytocin and thus accelerate cervix ripening (187). Similarly, a combination of acupressure, reflexology and aromatherapy can significantly shortened labor duration in nulliparous women (188). Beyond the special situation during parturition, frequent physical touch is associated with higher oxytocin levels for general population (189). In addition, massage (190) and running can also increase the secretion of oxytocin.

## 5 Perspectives

**Figure 2:**
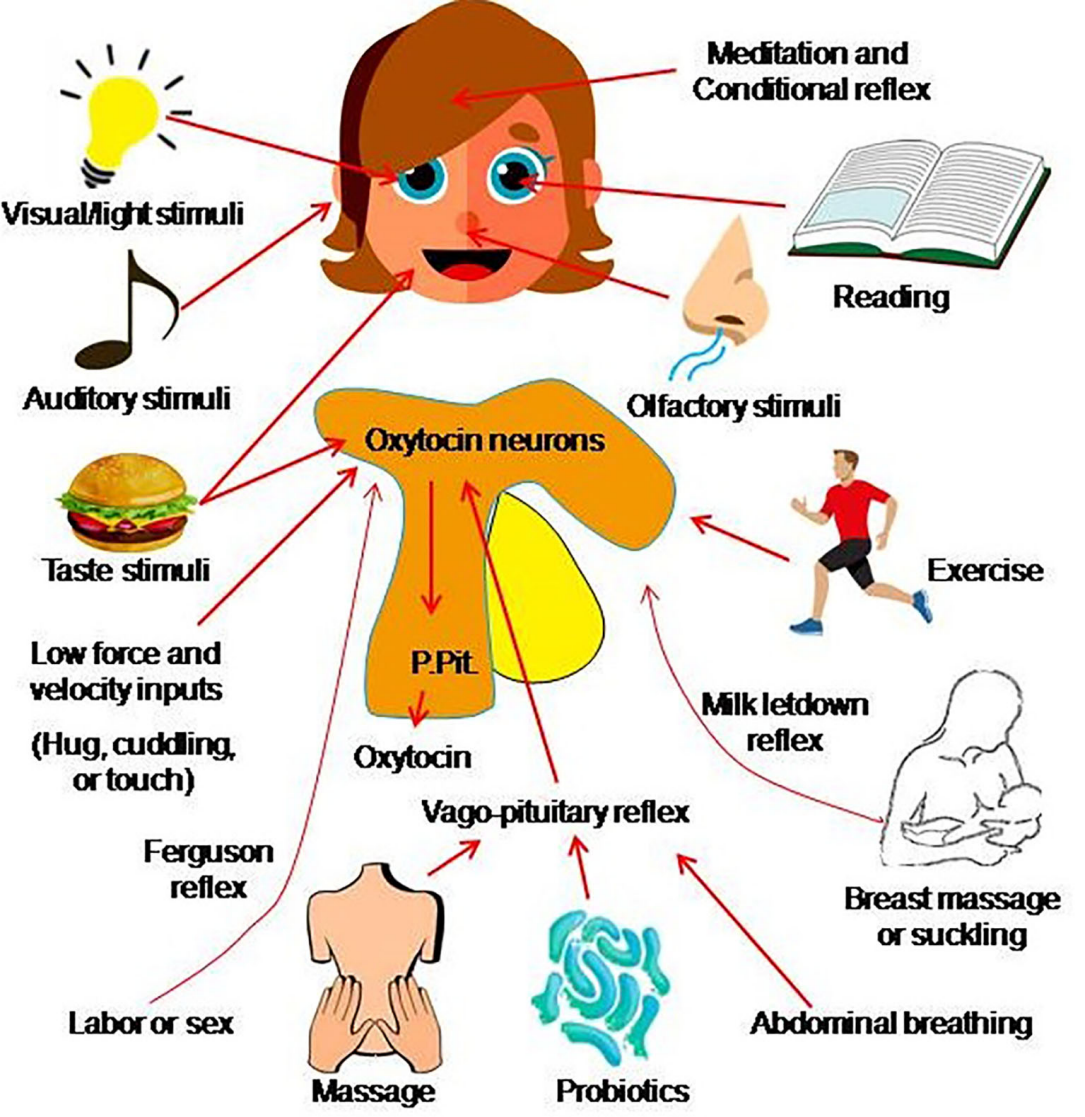
Approach of mobilizing endogenous oxytocin. Oxytocin secretion from the posterior pituitary gland (P.Pit) can be evoked by a variety of stimuli that activate oxytocin neurons in the hypothalamus and involve several pathways such as the sex-/labor-associated Ferguson reflex, the breastfeeding-associated let-down reflex (also called the milk-ejection reflex), the vago-pituitary reflex, as well as a conditioned reflex (acquired as the result of experience) and many other stimuli. The mechanisms underlying pathways from the stimulus to oxytocin secretion largely remain to be identified (the figure is an original drawing).

The pathogenesis of COVID-19 involves disorders in both innate immunity and adaptive immunity while SARS-CoV-2 entry is a prerequisite for disease onset. Thus, in the prevention of COVID-19, we should not only shield ourselves from the virus, cut potential transmission chains and receive timely vaccination, but also actively mobilize our own immunity to reduce the entry of SARS-CoV-2 into our body and its dissemination. Since oxytocin release can be evoked through numerous neurohumoral reflexes, mobilization of endogenous oxytocin secretion through physiological stimuli (**Figure 2**) is nevertheless an optimal approach to prevent COVID-19, independent of vaccination. More importantly, we need to set hands on clinical trials of oxytocin for controlling COVID-19. Such trials should fully balance the advantages and potential side effects of oxytocin application (123), consider the time- and dose-dependency of oxytocin/OTR signaling processes (157, 191), optimize the dosage form and administration approach, and combine oxytocin with application of inhibitors of SARS-CoV-2 replication and specific passive immunization (23). Thus, further exploring the preventive potential of mobilizing endogenous oxytocin secretion, executing intense clinical trials and applying exogenous oxytocin or its agonists are warranted in controlling COVID-19 and other viral diseases.

## Author Contributions

SW and FZ wrote the first draft and contributed to the key concepts; HZ, HY, YL and PW discussed the contents and drew the figures, VP and Y-FW conceived the study and edited the text. All authors contributed to the article and approved the submitted version.

## Funding

This work was supported by the National Key Research & Development Program of China (2017YFB0403805, FZ), the National Natural Science Foundation of China (grant No. 31471113, Y-FW), and Fund of the Ministry of Science and Technology of China (grant No. G2021011014L). VP is supported by a grant from the National Institute of General Medical Sciences of the National Institutes of Health (R01GM123971 to VP).

## Conflict of Interest

The authors declare that the research was conducted in the absence of any commercial or financial relationships that could be construed as a potential conflict of interest.

## Publisher’s Note

All claims expressed in this article are solely those of the authors and do not necessarily represent those of their affiliated organizations, or those of the publisher, the editors and the reviewers. Any product that may be evaluated in this article, or claim that may be made by its manufacturer, is not guaranteed or endorsed by the publisher.
